# Phase variation of manganese oxide in the MnO@ZnO nanocomposite with calcination temperature and its effect on structural and biological activities

**DOI:** 10.1038/s41598-023-48695-0

**Published:** 2023-12-06

**Authors:** Shatarupa Basak, Md Salman Haydar, Suranjan Sikdar, Salim Ali, Modhusudan Mondal, Ankita Shome, Kushankur Sarkar, Swarnendu Roy, Mahendra Nath Roy

**Affiliations:** 1 Department of Chemistry, University of North Bengal, Darjeeling, West Bengal 734013 India; 2 Department of Botany, University of North Bengal, Darjeeling, West Bengal 734013 India; 3 Department of Chemistry, Ghani Khan Choudhury Institute of Engineering and Technology (GKCIET), Malda, West Bengal 732141 India

**Keywords:** Biochemistry, Chemistry

## Abstract

Having powerful antibacterial and antioxidant effects, zinc oxide and manganese oxide nanomaterials are of great interest. Here we have synthesized manganese oxide decorated zinc oxide (MZO) nanocomposites by co-precipitation method, calcined at different temperatures (300–750 °C) and studied various properties. Here the crystalline structure of the nanocomposite and phase change of the manganese oxide are observed with calcination temperature. The average crystalline size increases and the dislocation density and microstrain decrease with the increase in calcined temperature for the same structural features. The formation of composites was confirmed by XRD pattern and SEM images. EDAX spectra proved the high purity of the composites. Here, different biological properties change with the calcination temperature for different shapes, sizes and structures of the nanocomposite. Nanomaterial calcined at 750 °C provides the best anti-microbial activity against *Escherichia coli, Salmonella typhimurium, Shigella flexneri* (gram-negative), *Bacillus subtilis* and *Bacillus megaterium* (gram-positive) bacterial strain at 300 µg/mL concentration. The nanomaterial with calcination temperatures of 300 °C and 450 °C provided better antioxidant properties.

## Introduction

Nowadays, nanotechnology is regarded as a validated state-of-the-art technology with a wide range of applications in the chemical, mechanical, pharmaceutical and food processing industries^1^. Biological, medicinal and environmental applications regularly make use of NPs^2–4^. Superior antibacterial activity has been discovered in metal oxide nanoparticles^5^. Metal oxide nanoparticles' antibacterial effectiveness is influenced by a several factors, including the type of microorganisms present, surface area, pH of the solution, particle size, crystallinity, capping/stabilizing agent, morphology, concentration and dosage. The bacteria's nanosize holes are easily penetrated by smaller nanoparticles with the right shape^6–8^. The ability of the production of reactive oxygen species, damage cell membranes, prevent uptake of critically important microelements by microbes, and several metals can exert direct genotoxic activity is the basis of metal oxide nanomaterials’ antimicrobial activity^9^. Numerous metal oxide nanostructures, including nickel (II) oxide, iron (II, III) oxide, silver oxide, titanium (IV) oxide, copper oxide, zinc oxide (ZnO), tin oxide and manganese (II, III) oxide nanomaterials (Mn_3_O_4_), have been studied closely as possible antibacterial systems^7,10–14^. In fact, antibacterial activity toward many hazardous species that are resistant to high temperatures and high pressures has been documented for ZnO nanostructures. ZnO nanostructures' increased surface area is what accounts for their higher antibacterial activity when compared to ZnO microstructures^15^. One of the most stable oxides, Mn_3_O_4_, is a highly effective catalytic component for the oxidative breakdown of volatile organic molecules. Additionally, medical research has shown that nanocrystalline Mn_3_O_4_ is a highly effective antibacterial and antioxidant system^16,17^. Because of their synergistic tendencies, several metal oxide hybrid systems have been thoroughly explored, including by our research group, as potential materials for antibacterial applications.

Chemical compounds in the form of nanoparticles are more readily absorbed by plants than other, more conventional, bulk forms because of their small size^18^. The interactions between NPs and plant cells, which result in both favourable and unfavourable morphological and physiological changes, are greatly influenced by the genotype, age, and developmental stage of the plants as well as the chemical composition, size, surface covering, shape, concentration, reactivity and mode of application of the nanoparticles^19–25^.

Metallic zinc is a crucial element for the overall growth and development of plants, playing a role in a number of physiological and enzymatic activities^26^. Zinc influences the capacity for water intake and transport, impacts seed viability and radical growth, and lessens the negative effects of heat, drought, or salt stressors. Additionally, zinc actively contributes to the synthesis of auxins and gibberellins^27^. In addition, ZnO NPs have a broad antifungal and antibacterial effect and could be used to prevent the spread of and treat infections caused by different plant diseases^28^.

For several functions, including photosynthesis and respiration, plants need the micronutrient manganese (Mn)^29^. Mn is abundant in the crust of the Earth and is easily oxidised to form around 30 different manganese oxides (MnO_x_)/hydroxides^30^. Nanoscale Mn is proven to be less phytotoxic and more efficient at reducing abiotic stressors in plants than typical bulk or ionic Mn molecules^31^.

In our present study, we synthesized manganese zinc oxide (MZO) nanocomposites, calcined at different temperatures within the range of 300–750 °C and studied their morphological, size and structural changes with the help of different characterization techniques. The biological efficacy of the synthesized complex was determined through antibacterial and antioxidant assays. The effectivity of the nanocomposites in the germination and growth of wheat seed (*Triticum aestivum*) was also studied in order to investigate their role in germination and subsequent plant growth.

## Materials and methods

### Chemicals

Manganese sulfate monohydrate (MnSO_4_⋅H_2_O), Zinc nitrate hexahydrate (Zn(NO_3_)_2_⋅6H_2_O), Ethylene glycol (EG) were provided by Merck (purity ≥ 98.9%). These substances were all put to immediate use without being further refined. All studies were conducted using dried and cleaned glassware.

### Synthesis of nanocomposites

10.7521 g of Zn(NO_3_)_2_^.^6H_2_O was added to 200 mL distilled water and stirred at 545 rpm for 40 min. 50 mL homogeneous solution of manganese sulfate (3.0003 g MnSO_4_⋅H_2_O in 50 mL distilled water and stirred for 20 min) was added dropwise to the solution and stirred for 20 min. Then 10 mL ethylene glycol is added and stirred for another 20 min. The pH of the solution was adjusted to ~ 9 using NaOH solution. Then the total solution was stirred for 2 h and dried to get nanopowder. After that, it was calcined at 300 °C, 450 °C, 600 °C, and 750 °C to get MZ 1, MZ 2, MZ 3 and MZ 4 respectively.

### Characterization

At room temperature, the structures of the synthesized nanomaterials were examined using an X-ray diffractometer (XRD) with a Bruker D8 Advance diffractometer and Cu Kα (λ = 1.5418 Å). A Perkin-Elmer Paragon 1000 FT-IR spectrometer was used to record FTIR spectra across a specific range of 450–4000 cm^−1^ at ambient temperature. To examine the surface morphologies of nanocomposites and average grain sizes, SEM pictures were acquired using a JEOL JSM-5800 working at an accelerating voltage of 10 kV. Energy dispersive X-ray spectroscopy (EDAX) (Hitachi SU8010 Series) was employed for the elemental analysis.

### Antibacterial activity

The antibacterial activity of differently calcinated MZO nanocomposites was assessed by adopting the disc diffusion method^32,33^. In this study, the antibacterial activity of test compounds was evaluated against five bacterial strains including *Escherichia coli, Salmonella typhimurium, Shigella flexneri* (gram-negative), *Bacillus subtilis* and *Bacillus megaterium* (gram-positive). A loopful of each bacterial strain was inoculated in sterile nutrient broth and incubated for 24 h at 37 °C to get fresh and viable bacterial inoculum. After evenly mixing the test organism (200 µL of bacterial suspense) in a nutrient agar plate, it was allowed to harden. The test compounds dissolved in DMSO to obtain a concentration of 100, 200 and 300 µg/mL. Sterile paper disks impregnated with the test compounds were placed on the inoculated plates using sterile forceps and the plates were incubated at 37 °C for 24 h. The bactericidal effect of different grades of MZO nanocomposites was initially assessed using the highest concentration of the tested sample (300 µg/mL) and only the effective grade of nanocomposites was further tested in different concentrations. The diameter of the inhibition zone was measured in millimeters using a ruler.

### Antioxidant activity

The antioxidant propensity of MZO nanocomposites (synthesized using different calcination temperatures) was investigated following the colorimetric decolorization technique against ABTS^+^, DPPH, superoxide and nitric oxide radical according to the protocol mentioned previously^34,35^. The antioxidant efficacy of the tested samples was determined by calculating the inhibition percentage and the IC_50_ values, which represent the theoretical concentration of the tested sample at which 50% of the free radicals are scavenged, were used to express the antioxidant activity of the sample.

$$Inhibition\,percentage=\left[\left(\frac{Ac-As}{Ac}\right)\times 100\right]$$

where, Ac is the absorbance of the control and As is the absorbance of the tested sample.

### Wheat seed germination and plant growth study

The ‘Sonalika’ cultivar of wheat was collected from National Seeds Corporation Limited, New Delhi, India. Collected seeds were surface sterilized with 4% sodium hypochlorite and then 500 µg/mL of different grades of synthesized nanocomposites (i.e. MZ 1, MZ 2, MZ 3 and MZ 4) were applied through seed priming. The initial germination assay was carried out in Petri plates (200 seeds were taken) and based on germination percentage optimal grades of MZO nanocomposites were chosen. After 3 days of germination, germinated seeds of optimal grade and control were transferred onto another Petri plate and seedlings were grown on nutrient-free sand under a natural photoperiod. The transplanted seedlings were maintained up to 20 days after transplantation (DAT) and the optimal dose of MZO nanocomposite was applied two times (in solution form) between the period (7 DAT and 14 DAT). Growth, biomass, and biochemical parameters were evaluated after harvesting the seedlings at 20 DAT. The shoot length was measured using a centimeter scale, whereas biomass was determined by using digital weight balance (Quintix 224, Sartorius Lab Instruments).

### Evaluation of biochemical content of wheat seedlings

Chlorophyll content was estimated following the well-known methodology of Arnon (1949)^36^. The protocol mentioned previously^37^ was used to determine the total sugar content of the seedlings. After crushing the seedlings in potassium-phosphate buffer (pH-7.0), the estimation of protein content was done by another renowned method prescribed by Lowry et al.^38^.To estimate the total phenol content, the procedure provided by Kadam et al. was followed with minor modifications^39^.

### Data analysis and program used

Growth parameters and plant biomass were calculated by taking readings from 20 replicas. For plant biochemical, antioxidant, and antimicrobial study three replications were taken into consideration and results were presented as average with standard deviation. Statistical differences were carried out by Tukey’s HSD test at p ≤ 0.05 (for the germination test) and a two-tailed t-test at 95% confidence level (plant growth and biochemical parameters).

## Result and discussions

### Characterization of the nanocomposites

**Figure 1:**
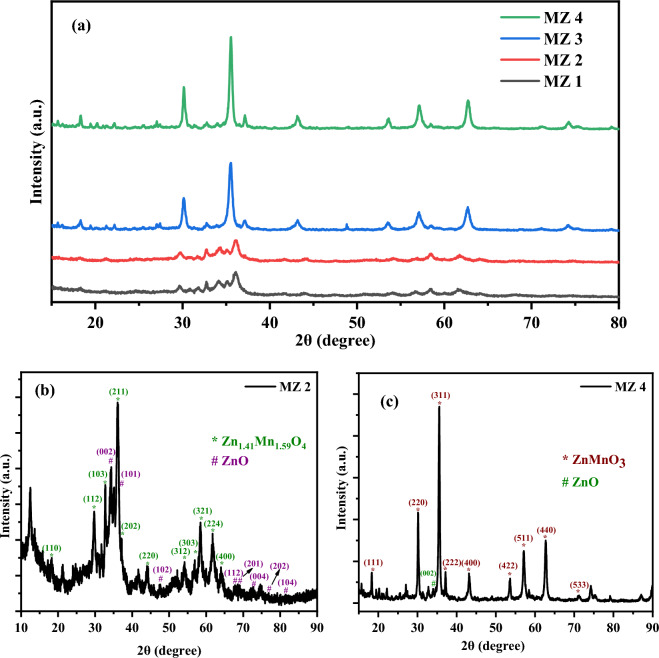
X-ray diffraction pattern of **(a)** all nanocomposites, **(b)** MZ 2 and **(c)** MZ 4.

X-ray diffraction (XRD) is a powerful tool that is frequently used and widely recognized for both qualitative and quantitative studies of crystalline phases present in materials^40^. A careful examination of the diffraction patterns can reveal much more information, including the characterization of solid substances, crystallite size and shape, crystal orientation, and internal elastic strains/stresses at various levels^41^. From Fig. 1a we can see that, the XRD pattern of MZ 1 and MZ 2 are almost the same i.e. at 300 °C and 450 °C the nanocomposite possesses the same crystal structure. The diffraction peak positions at 2θ = 18.31, 29.75, 32.22, 36.05, 37.08, 44.09, 54.09, 56.96, 58.21, 61.76 and 64.12 correspond to the (110), (112), (103), (211), (202), (220), (312), (303), (321), (224) and (400) planes respectively shown in Fig. 1b which matched to the Zn_1.41_Mn_1.59_O_4_ phase. The XRD pattern belongs to the tetragonal structure and space group *I41/amd* (141) with a = b = 5.80390 Å and c = 8.79830 Å matching with the PDF code no. 00-061-0715^42^. The other peaks appeared at 2θ = 34.56, 36.49, 47.57, 67.86, 68.99, 72.68, 76.81 and 81.51 indicating the (002), (101), (102), (112), (201), (004), (202) and (104) planes respectively predicting the hexagonal structured of zinc oxide (ZnO) with *P63mc* space group and lattice parameters were found to be a = b = 3.3420 Å and c = 5.176 obtained from literature studied with PDF code no. 00-001-1136^43^. In MZ 1 and MZ 2 there is the existence of two different phases in both and the peak intensity slightly increases from MZ 1 to MZ 2, i.e. with an increase in calcination temperature.

Interestingly the XRD pattern of MZ 3 and MZ 4 were also the same which confirmed the same crystal structure of the nanocomposites at calcinations temperature 600 °C and 750 °C. Here (Fig. 1c) the diffraction peaks are appeared at 2θ = 18.34, 30.14, 35.56, 37.15, 43.14, 53.62, 57.13, 62.71, 71.25 correspond to the (111), (220), (311), (222), (400), (422), (511), (440) and (533) planes respectively assigned to the cubic phase of ZnMnO_3_ (JCPDS card no. 19–1461)^44^. The peak appears at position 34.02 corresponding to the (002) plane of ZnO confirming the different phases present in the nanocomposites^43^. The sharp peak of MZ 3 and MZ 4 confirms the high crystalline nature of the prepared sample and peak intensity increases with the increase in temperature.

The average crystalline size can be calculated by using Scherer’s equation

2 $$\mathrm{D }= \frac{0.9\lambda }{\beta cos\theta }$$

where, D is the average crystalline size, λ is the wavelength of the radiation (0.154 nm), θ is the angle of diffraction, β is the full width at half maximum intensity^45^. Also dislocation density (δ), crystallinity and microstrain (ɛ) can be calculated using the following equations^46^:

3 $$\updelta =1/{D}^{2}$$

4 $$\upvarepsilon =\frac{\beta cos\theta }{4}$$

5 $$\mathrm{Crystallinity }= \frac{area\,of\,the\,crystalline\,peaks}{area\,of\,all\,peaks (crystalline+amorphous)}$$

The average crystalline sizes of MZ 1 and MZ 2 are 19.69 nm and 26.41 nm, i.e., size increases with an increase in calcination temperature. The crystallinity was increased from MZ 1 to MZ 2 (46.38% for MZ 1 and 70.41% for MZ 2) but dislocation density and micro-strain decreased from MZ 1 to MZ 2 (Tables S1 and S2). Again, the calculated average crystalline sizes of MZ 3 and MZ 4 are 20.98 nm and 26.12 nm respectively. Here, size and crystallinity increase (crystallinity 66.78% for MZ 3 and 78.91% for MZ 4) as well as dislocation density and microstrain decrease with an increase in calcination temperature from 600 to 750 °C (Tables S3 and S4).

**Figure 2:**
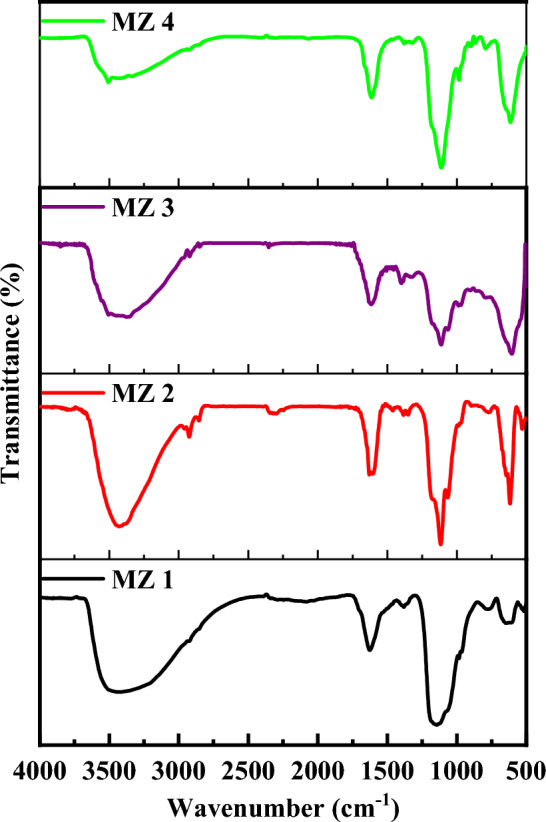
FTIR spectra of MZ 1, MZ 2, MZ 3 and MZ 4.

Figure 2, represents the FTIR spectra of the nanocomposites, the broad peak for all of the nanocomposites at wavenumber 3300–3600 cm^−1^ is caused by the presence of O–H stretching arising from the H_2_O molecule^45^. Two small, sharp peaks at 2923 cm^−1^ and 2854 cm^−1^ are the asymmetric stretching and vibrations of –CH_2_– present in aliphatic chains^47^. At high calcination temperatures, such peaks get less intense. The peak arises in the range of 1626–1630 cm^−1^ due to the O–H bending vibrational mode for moisture^45^. As a result, the intensity of the peak is decreased as enhanced the calcination temperatures of the nanocomposites. The peak is visible at 1384 cm^−1^ due to the C–H bending vibrational bands^48^. Peaks for the C–O stretching vibration are found to be in the frequency range of 1110–1115 cm^−1^^49^. The stretching frequency in the range of 500 to 1000 cm^−1^ corresponds to the metal–oxygen bond present in the prepared nanocomposites. It is normal to see the unique stretching vibration frequency of Zn–O at 915 cm^−1^ and 750 cm^−1^ for the presence of Zn–O bonds^50^. The distinct Zn–O peaks in composites appeared at 987 cm^−1^ and 793 cm^−1^, respectively, due to a shift to a higher frequency^51^. The Mn–O bond is what causes MZ 1 and MZ 2 to peak at 530 cm^−1^^52^. The peak at about 613 cm^−1^ for MZ 3 and MZ 4 confirms the presence of the (Mn–Zn)–O stretching mode. Here, the presence of M–O and M–O–M bonds demonstrates that ZnMnO_3_ was formed^53^.

**Figure 3:**
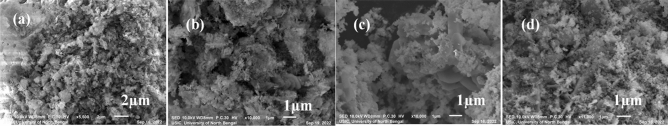
SEM images of (**a**) MZ 1, (**b**) MZ 2, (**c**) MZ 3 and (**d**) MZ 4.

SEM is a technique that can be used to evaluate the surface texture^54^. In Fig. 3 the SEM images of the composites, the presence of two different instinct shapes of particles confirms the formation of composites. The porous nature and morphology of the samples at various synthesized temperatures from 300 to 750 °C are shown by the SEM pictures in Fig. 3. The morphology of the samples exhibits a noticeable shift in the SEM pictures of MZ 2 to MZ 3, i.e. increasing calcination temperatures of the synthesized nanocomposites. These may be due to the formation of different phase of manganese oxides. Quite large low-density agglomerates are generated, according to SEM examination. Continuous agglomeration of small particles occurs at higher calcining temperatures.

**Figure 4:**
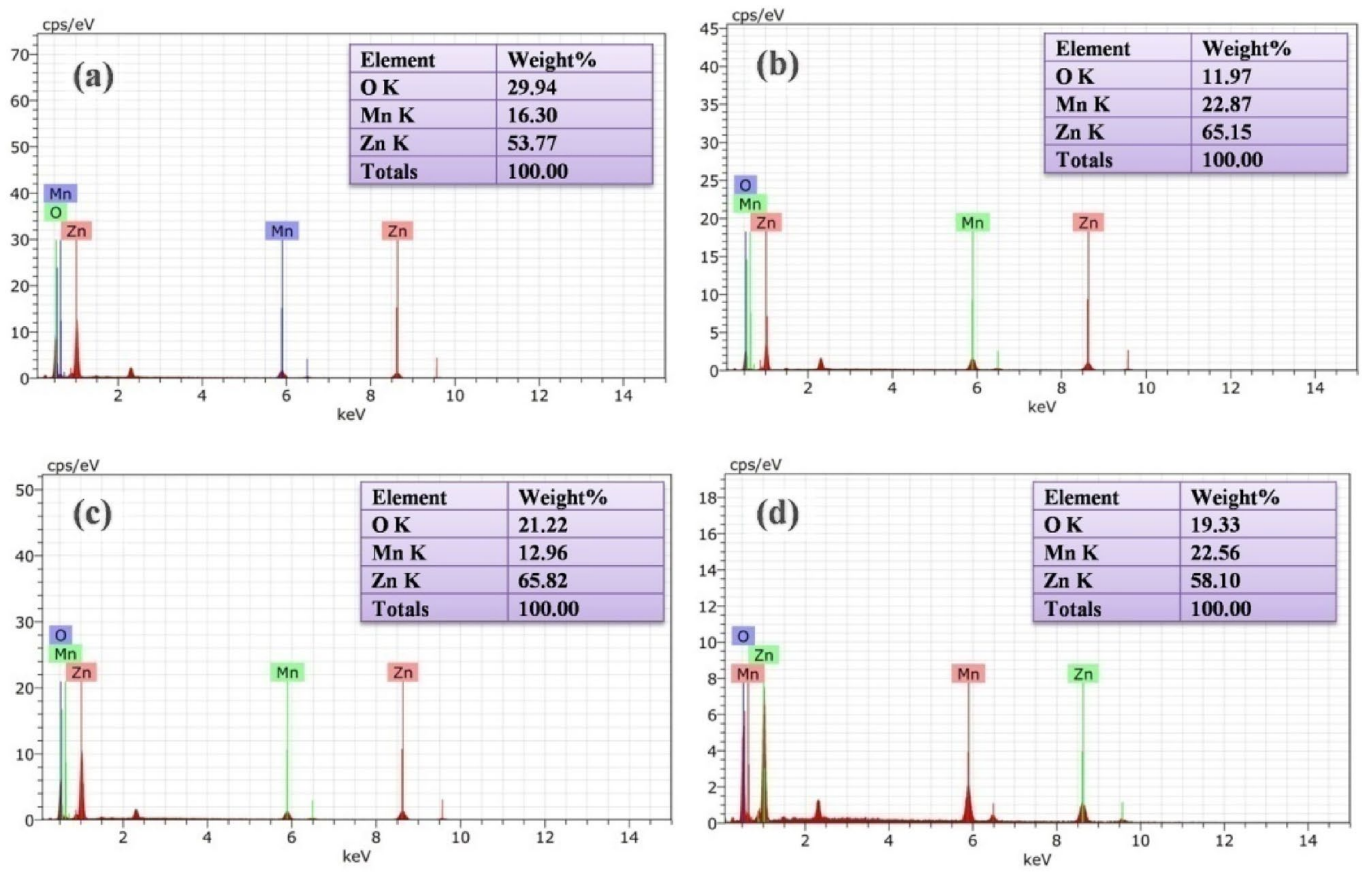
EDS spectra of (**a**) MZ 1, (**b**) MZ 2, (**c**) MZ 3 and (**d**) MZ 4.

Using the EDAX spectrum, the elemental analysis of MZ 1, MZ 2, MZ 3 and MZ 4 were examined as depicted in Fig. 4. The existence of peaks attributable to manganese, zinc and oxygen was visible in the EDAX spectrum, which demonstrated that carbon and other organic residue had been removed from the template in the calcined samples. Zn, Mn and O are the sample’s main constituents, according to the strong signal at 8.6 and 1.0 keV for Zn; 0.6 and 5.9 keV for Mn and 0.5 keV for O. The purity of the synthesized composites were amply supported by the absence of peaks caused by other components. Here the weight % of the Zn and Mn ratio are almost the same and matched approximately with the ratio taken at the time of synthesis.

### Antibacterial effect of MZO nanocomposites

**Figure 5:**
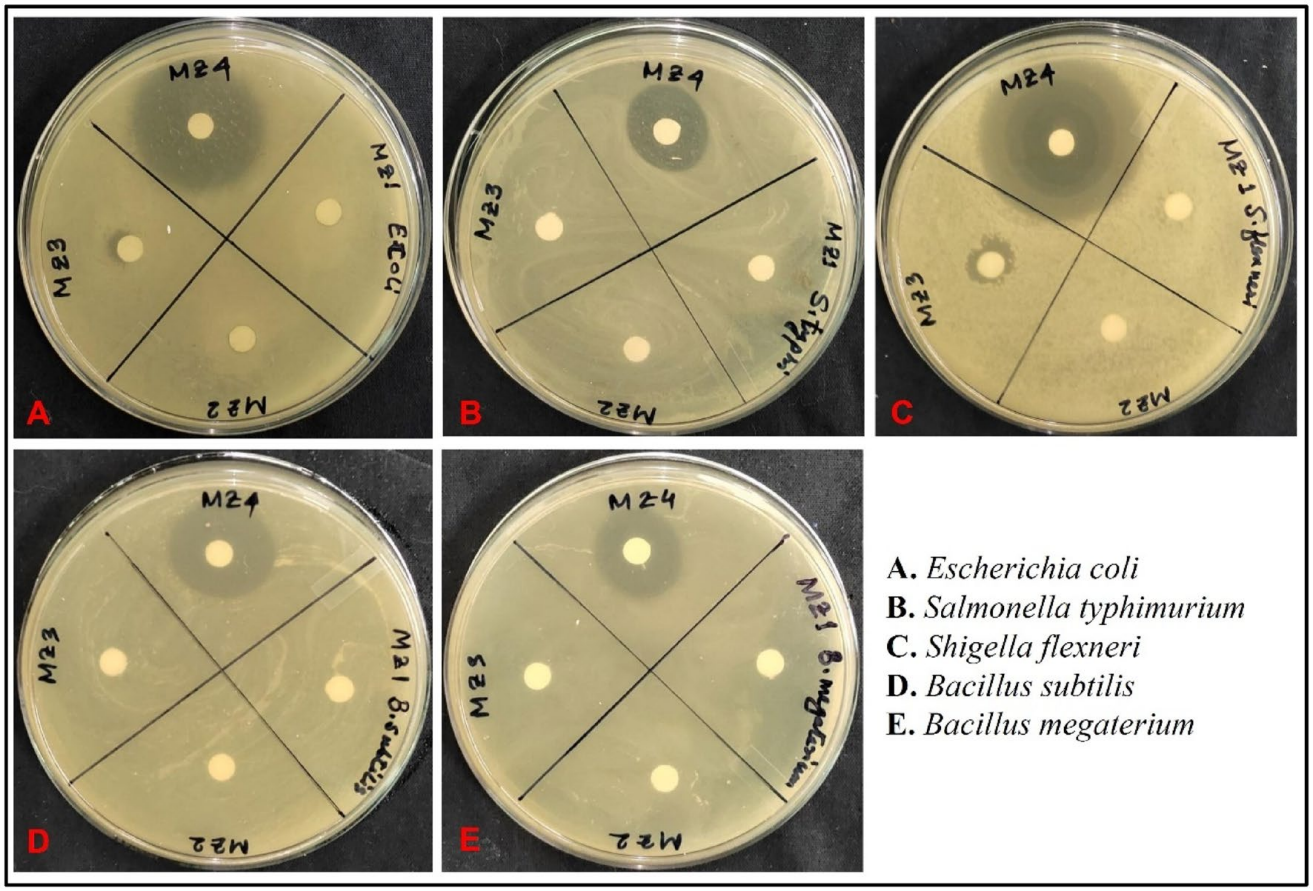
Initial screening of antibacterial activity of MZ 1, MZ 2, MZ 3 and MZ 4 at 300 µg/mL against the tested microorganism.

The results of the present study demonstrate the antibacterial activity of MZO nanocomposites against five different bacterial strains (Fig. 5). From the initial screening with the highest concentration of sample, it was found that MZ 4 was the best (Fig. 5). The synergistic effect of different phases of manganese oxide and ZnO nanoparticles in MZ 4 may contribute to its enhanced antibacterial activity. Thus, MZ 4 was further tested against the bacterial strains at three different concentrations (100, 200 and 300 µg/mL). It highlighted the importance of optimizing the calcination temperature of nanocomposites to obtain the best antibacterial activity^55^. In the present study, the superior activity of MZ 4 could be attributed to the changes in the morphology, crystalline structure, and composition of the nanocomposites that occurred during calcination at 750 °C. The attributed antibacterial activity may be due to the generation of reactive oxygen species (ROS) by the nanocomposites, which caused damage to the bacterial cell membrane and cytoplasmic structures. There are such reported facts in a manganese oxide and zinc oxide nanocomposites study^56^.

**Figure 6:**
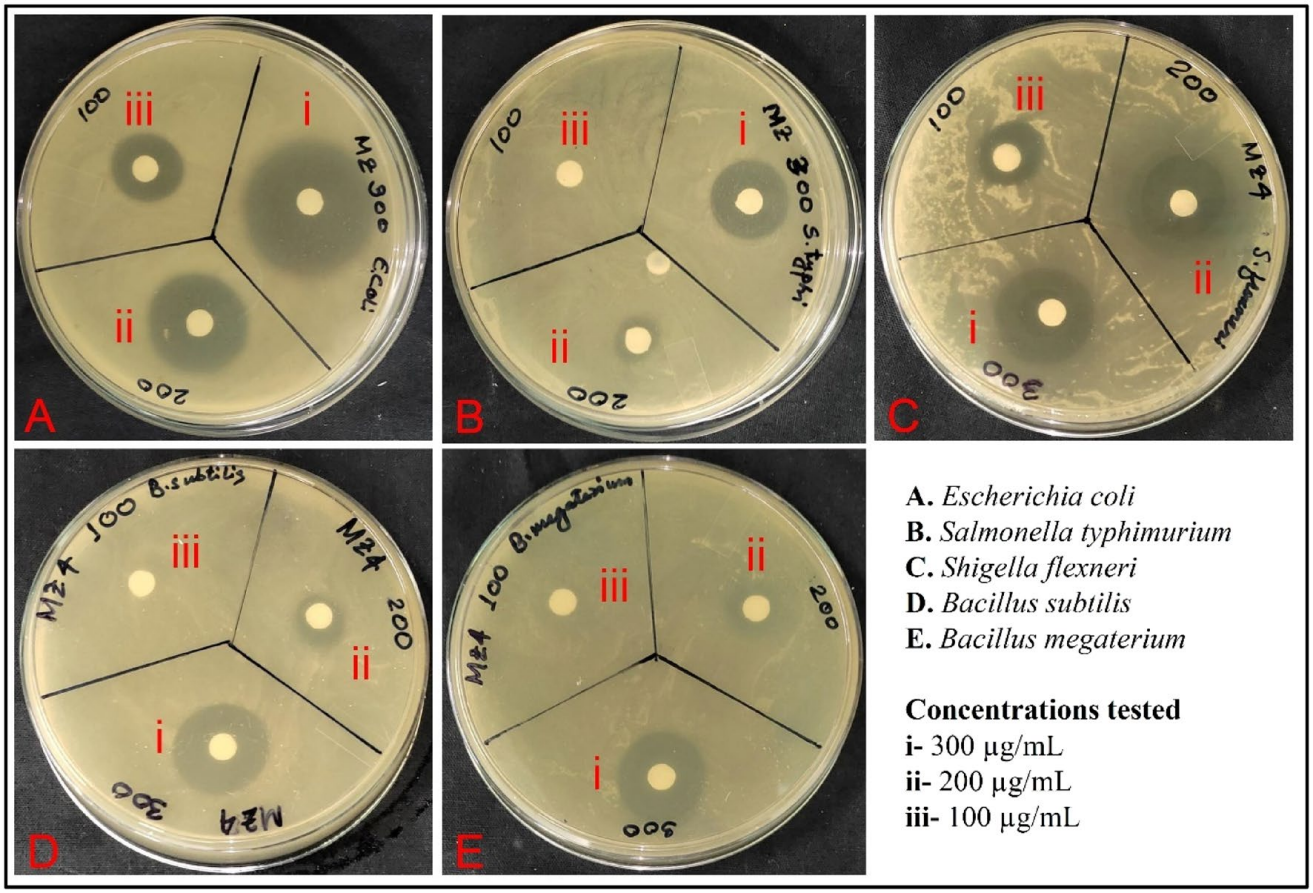
Antibacterial activity of MZ 4 against the tested bacterial strain.

**Figure 7:**
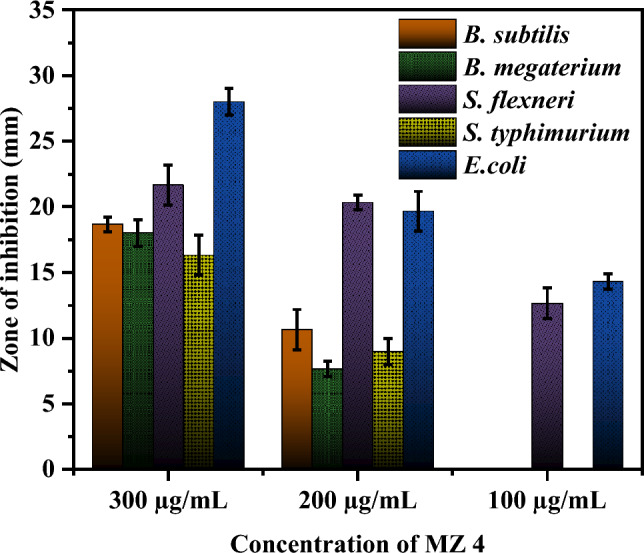
Zone of inhibition of MZ 4 against different bacterial strain at different concentration.

The antibacterial activity of the nanocomposites was tested at different concentrations, and it was observed that the zone of inhibition increased with an increase in concentration. This trend was observed for all the bacterial strains tested. Several previous reports support this concentration-dependent antibacterial activity against different bacterial strains^57^. The highest zone of inhibition was observed for *Escherichia coli* (28 ± 1.00) at a concentration of 300 µg/mL (Figs. 6, 7). *Shigella flexneri* was also found to be susceptible to the nanocomposites, whereas *Salmonella typhimurium* among gram negatives and *B. megaterium* among gram positives were the least susceptible, with no zone of inhibition observed at a concentration of 100 µg/mL (Table S5). In the current study, it was observed that the gram-negative strains showed higher susceptibility than the positive ones. The observed variation in antibacterial activity could be attributed to the differences in the cell wall structures and physiological characteristics of the bacterial strains. The findings of the present study are consistent with previous studies^58^.

### Antioxidant activity of MZO nanocomposites

**Figure 8:**
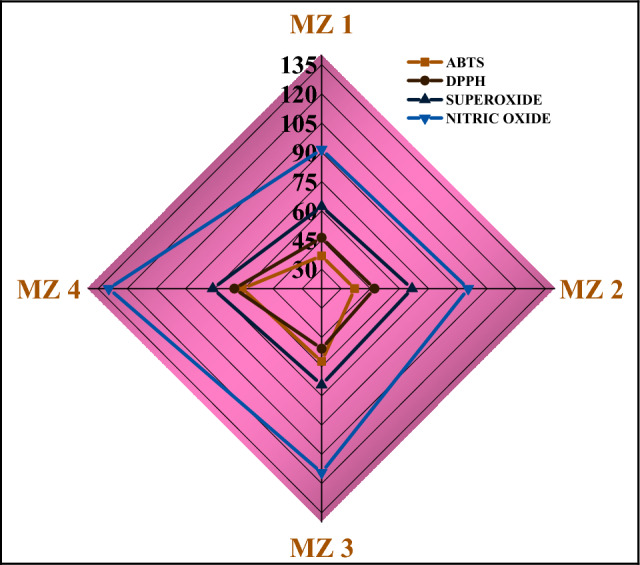
Radar plot for comparative effect of MZ 1, MZ 2, MZ 3 and MZ 4 against different free radicals. (scale bar represents the IC 50 value in µg/mL).

Several types of chemical entities with one or more unpaired electrons are known as free radicals. These free radicals destroy other molecules by extracting their electrons to become stable. More importantly, these free radicals are generated all the time inside the body system as a by-product of several important reactions. In the current investigation, antioxidant activity was expressed as an IC_50_ value, and presented in Table S6. The high IC_50_ value of a sample indicates comparatively low antioxidant activity. From the presented table and Fig. 8, it was observed that in maximum cases against all the studied free radicals no such strong differences in antioxidant activity were observed among MZ 1 (IC_50_ values are 36.845 ± 0.126 µg/mL for ABTS, 46.127 ± 1.622 µg/mL for DPPH, 62.194 ± 0.180 µg/mL for superoxide and 91.487 ± 5.754 µg/mL for NO) and MZ 2 (36.974 ± 0.481 µg/mL for ABTS, 47.145 ± 0.681 µg/mL for DPPH, 66.456 ± 0.110 µg/mL for superoxide and 95.347 ± 6.459 µg/mL for NO). Furthermore, with increasing calcination temperature, the antioxidant efficacy of all the studied samples gradually decreases. These may be due to the increase in particle size and decrease in active surface to capture the radicals^59^. It is reported that the best antioxidant activity of iron oxide nanoparticles calcined for 2–3 h, maintaining the temperature in the range of 200–300 °C, which coincides with our present study^60^. They further reported a strong reduction in antioxidant activity above 500 °C, which also supported our findings. Overall results depicted that synthesized MZO nanocomposites demonstrated the highest activity against ABTS^+^ radical and the least against nitric oxide radical.

### Effect of MZO nanocomposites on wheat seed germination and seedling growth

**Figure 9:**
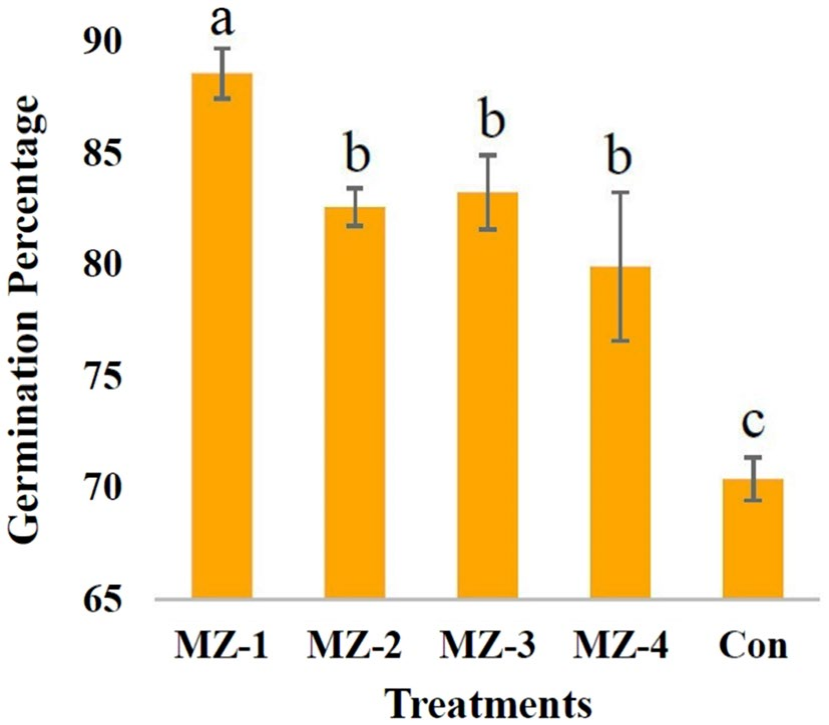
Germination percentage of wheat seed as influenced by different types of MZO nanocomposites. The vertical bar above column represents the standard deviation (n = 3). Different letters (a, b, c etc.) indicated that they are statistically different at p ≤ 0.05 following Tukey’s HSD test.

The process of seed germination is fundamental to the emergence of new plants, but it is of great significance as it ultimately determines factors such as crop quality and yields. A seed germination study was carried out by taking 200 seeds and it was found that MZ 1 treatment was the most effective that can germinate almost 89% of seeds. Though among other grades of MZO nanocomposites (i.e. MZ 2, MZ 3 and MZ 4), no such strong statistical variability (at p ≤ 0.05) in seed germination was observed (Fig. 9). From the germination assay, among different grades of nanocomposites MZ 1 was chosen as it demonstrates 25.87% more seed germination capability than the control, and for that reason, further seedling growth study was carried out with this particular treatment (MZ 1) in comparison to control.

**Figure 10:**
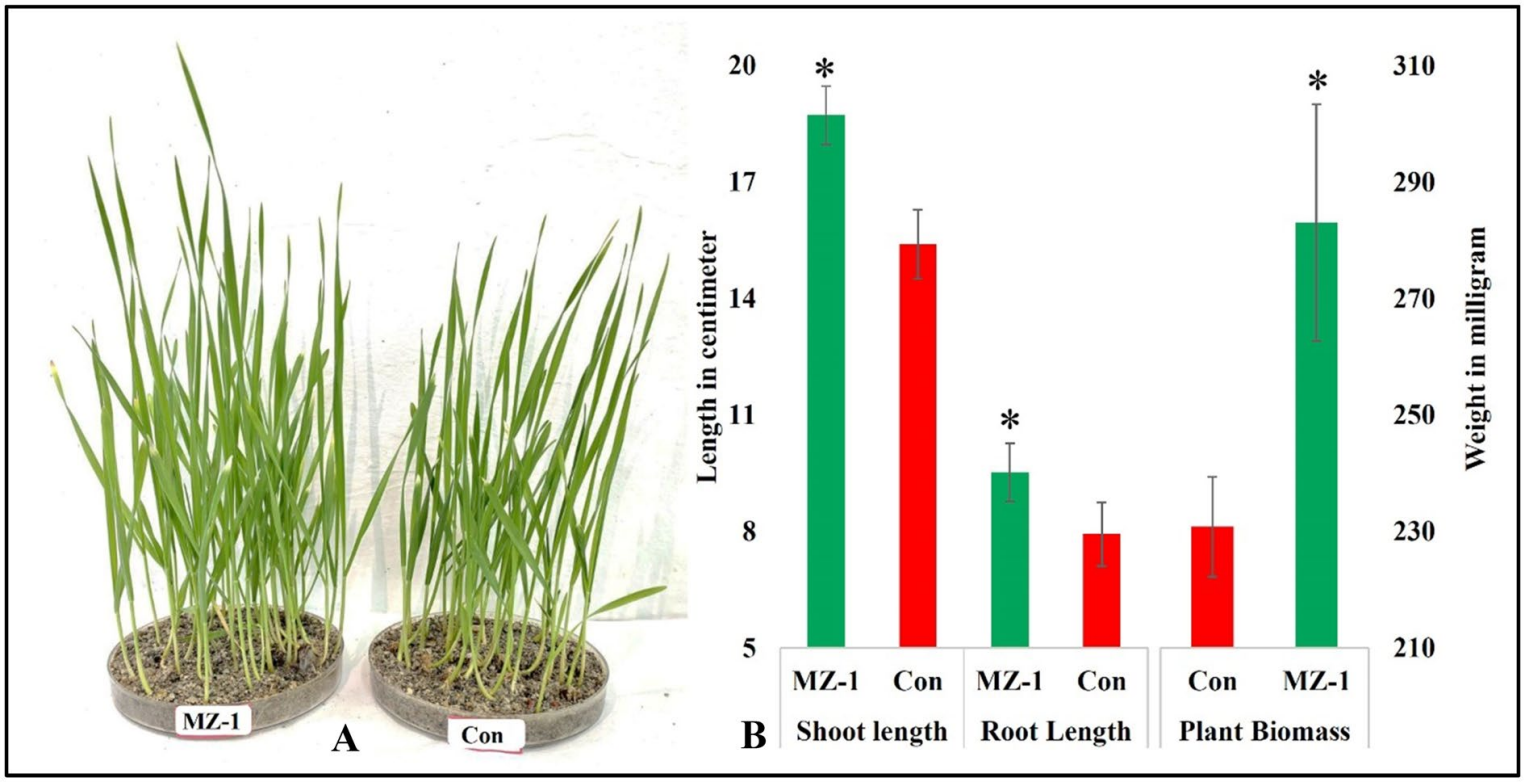
Phenotypic appearance **(A)** and growth attributes **(B)** of treated and control and treated wheat seedlings. The vertical bar above the column represents the standard deviation (n = 20). Asterisk: symbol indicated that they are statistically significant from their respective control at 95% confidence level as observed through two-tailed t-test.

On evaluating phenotypic appearance, the same trends of results were found, where almost every growth parameter such as shoot height, root length, and plant biomass identified to be strongly influenced by MZ 1 treatment (Fig. 10). This particular treatment increased seedling shoot height and root length by 21.63% and 20% respectively in comparison to control seedling. Root length improvement as observed through seeds priming with MZ 1 nanocomposites may be attributed to the activation of cell cycling and respiration during the priming process. This activation, along with the translocation of assimilated nutrients and withering of the seed coat, leads to faster root emergence, as described in previous studies^61^. Root parameters improvement through MZO nanocomposites might be due to the influence of outside factors like the presence of mineral nutrition, which regulates numerous morpho-physiological processes. Plant biomass was also enhanced by 23% from control by application of MZ 1 through seed priming and subsequent irrigation.

### Effect of MZO nanocomposites on wheat seedling biochemical attributes

The most important factors influencing plant growth and development include the rate of photosynthesis, the assimilation of sugars, and the amount of protein present. The application of MZO nanocomposites was found to have a moderate to strong effect on biochemical parameters such as chlorophyll, sugar, protein, and phenol content. In contrast to control seedlings, those treated with MZ 1 exhibited a 20.83% increase in chlorophyll content. Several studies that have been published claimed that the use of nanoparticles significantly increased the amount of chlorophyll by creating more light-harvesting complexes and absorbing the most amount of light energy, which in turn increased of the rate photosynthesis^62,63^. Though the excessive application of zinc was reported to inhibit chlorophyll synthesis by interfering with the iron metabolism of plants^64^, in the present study no such effect of chlorophyll retardation was observed. It might be due to the co-application of Zn and Mn working in a balanced system as Mn has an immense role in chlorophyll biosynthesis, and oxygen evolution during photosynthesis and is found to be involved in the chloroplast^65,66^. Optimal grade MZO nanocomposites (MZ 1) treated wheat seedlings had the highest total carbohydrate content, surpassing the control by 31.63%. Indeed, sugars are essential as regulatory molecules in the central signaling system that control the expression of genes involved in metabolism, growth, and development^67^. The application of MZ 1 promisingly enhances (39.47% over control) the protein content of the treated wheat seedling. The amount of protein in leaves is extremely important and plays a significant function in plant growth, reproduction, and ultimately grain yield^68^. A lot of published works reported the role of micronutrients like Fe, Mn, Zn and Cu (applied in their nano form) in the improvement of plant protein content and subsequent growth and development^37,69,70^. On analysis of total phenol content, it was observed that there was no such strong statistical difference between control and treated seedlings (Table 1).

**Table 1:** Various biochemical attributes of treated and control wheat seedlings.

Treatments	Chlorophyll (mg/g FWT)	Protein (µg/mg FWT)	Sugar (µg/mg FWT)	Phenol (µg/mg FWT)
MZ 1	2.916 ± 0.043*	15.93 ± 0.604*	12.976 ± 0.291*	0.940 ± 0.026
Con	2.433 ± 0.083	11.48 ± 0.112	9.844 ± 0.135	0.923 ± 0.031

Results were presented as mean ± standard deviation, whereas ‘*’ symbol indicated that they are statistically significant from their respective control at 95% confidence level as observed through two-tailed t-test.

## Conclusion

In our work, we study the effects of calcination temperature on structural, anti-bacterial, anti-oxidant and seed germination processes. The prepared nanocomposites were characterized using different techniques like XRD, FTIR, SEM and EDX. The characterization results showed that with the variation of calcination temperatures, the synthesized nanocomposite MZO transformed into different structures and sizes. At low calcination temperatures the composite exists as Zn_1.41_Mn_1.59_O_4_/ZnO and is converted into ZnMnO_3_/ZnO at higher calcination temperatures. The bioactivities are greatly influenced by the varied temperature. For this reason, the bioactivities of the composite changes with the different calcination temperatures. Composite shows the best antibacterial at 750 ^0^C and best antioxidant and seed germination properties at 300 ^0^C indicating the temperature controlling activities are pronouncedly found in the nanocomposites giving a new way for future research.

### Supplementary Information

Supplementary Tables.

## Data Availability

Data will be made available on request to the corresponding author.
