# Supplementary material for: Phase variation of manganese oxide in the MnO@ZnO nanocomposite with calcination temperature and its effect on structural and biological activities
Source: Sci Rep. 2023 Dec 6;13:21542. doi: 10.1038/s41598-023-48695-0 (PMC10700637; doi:10.1038/s41598-023-48695-0)
Supplement: Supplementary file 1 — Supplementary Tables. [file 41598_2023_48695_MOESM1_ESM.docx]

**Phase Variation of Manganese Oxide in the MnO@ZnO Nanocomposite with Calcination Temperature and its Effect on Structural and Biological Activities**

Shatarupa Basak^a^, Md Salman Haydar^b^, Suranjan Sikdar^c^, Salim Ali^a^, Modhusudan Mondal^a^, Ankita Shome^a^, Kushankur Sarkar^b^, Swarnendu Roy^b^, Mahendra Nath Roy^a,d^*

^a^Department of Chemistry, University of North Bengal, Darjeeling-734013, West Bengal, India.

^b^Department of Botany, University of North Bengal, Darjeeling-734013, West Bengal, India

^c^Department of Chemistry, Ghani Khan Choudhury Institute of Engineering and Technology (GKCIET), Malda-732141, West Bengal, India.

***Corresponding Author**:

Mahendra Nath Roy: mahendraroy2002@yahoo.co.in, vcapduniversity@gmail.com.

**Number of pages: 03**

**Number of Tables: 06**

**Table S1:** Size and other lattice parameters of the MZ 1 nanocomposites

| 2θ (degree) | FWHM (degree) | Crystalline size (nm) | Average Crystalline size (nm) | Micro-strain (ɛ × 10^-3^) | Average Micro-strain (ɛ × 10^-3^) | Dislocation Density (δ × 10^-3^) (nm^-2^) | Average Dislocation Density (δ × 10^-3^) (nm^-2^) |
| --- | --- | --- | --- | --- | --- | --- | --- |
| 12.4564 | 0.41641 | 18.96544113 | 19.69664493 | 1.806207289 | 1.891880674 | 2.780187604 | 5.291002379 |
| 18.316 | 0.19641 | 39.93190133 |  | 0.846076284 |  | 0.62713353 |  |
| 24.6361 | 0.31395 | 24.72176743 |  | 1.338328826 |  | 1.63621725 |  |
| 29.6513 | 0.42794 | 17.94600918 |  | 1.805078075 |  | 3.105018764 |  |
| 30.8795 | 0.19586 | 39.0972484 |  | 0.823758739 |  | 0.654195591 |  |
| 31.8518 | 0.39681 | 19.25195008 |  | 1.664954173 |  | 2.698053432 |  |
| 32.7218 | 0.23146 | 32.93267247 |  | 0.969038905 |  | 0.922032119 |  |
| 34.1291 | 0.83413 | 9.104743606 |  | 3.479345397 |  | 12.06325642 |  |
| 35.1526 | 0.51732 | 14.63971066 |  | 2.151856826 |  | 4.665895557 |  |
| 36.0994 | 0.73664 | 10.25376781 |  | 3.056021569 |  | 9.511150239 |  |
| 43.878 | 0.67475 | 10.9210533 |  | 2.730943116 |  | 8.384379866 |  |
| 47.4091 | 0.46896 | 15.51099172 |  | 1.873585467 |  | 4.156433801 |  |
| 50.7611 | 0.29518 | 24.3157158 |  | 1.163652365 |  | 1.691320382 |  |
| 54.0875 | 0.69683 | 10.1540653 |  | 2.708045594 |  | 9.698846734 |  |
| 56.6462 | 0.54603 | 12.80744197 |  | 2.097286427 |  | 6.096424599 |  |
| 58.5141 | 0.5652 | 12.26270835 |  | 2.151557943 |  | 6.650085079 |  |
| 59.9982 | 0.22712 | 30.29243789 |  | 0.858237182 |  | 1.089761719 |  |
| 61.5335 | 0.84169 | 8.110094174 |  | 3.155677207 |  | 15.20366198 |  |
| 64.041 | 0.43522 | 15.47638549 |  | 1.610087836 |  | 4.175042707 |  |
| 68.2374 | 0.79559 | 8.266690917 |  | 2.87390675 |  | 14.63310874 |  |
| 74.7878 | 0.16323 | 38.66674642 |  | 0.565848192 |  | 0.668843852 |  |

**Table S2:** Size and other lattice parameters of the MZ 2 nanocomposites

| 2θ (degree) | FWHM (degree) | Crystalline size (nm) | Average Crystalline size (nm) | Micro-strain (ɛ × 10^-3^) | Average Micro-strain (ɛ × 10^-3^) | Dislocation Density (δ × 10^-3^) (nm^-2^) | Average Dislocation Density (δ × 10^-3^) (nm^-2^) |
| --- | --- | --- | --- | --- | --- | --- | --- |
| 12.482 | 0.54333 | 14.53482525 | 26.41430609 | 2.356674085 | 1.737802813 | 4.733478085 | 4.176769641 |
| 18.2648 | 0.43551 | 18.01012352 |  | 1.876183427 |  | 3.082950965 |  |
| 21.2586 | 0.33633 | 23.21521767 |  | 1.442335575 |  | 1.85547232 |  |
| 29.7281 | 0.62451 | 12.29516111 |  | 2.633754912 |  | 6.61502595 |  |
| 32.7474 | 0.40259 | 18.93265171 |  | 1.685387439 |  | 2.789825946 |  |
| 34.3594 | 0.53615 | 14.15618622 |  | 2.235019047 |  | 4.990079514 |  |
| 35.127 | 0.7164 | 10.57223739 |  | 2.980165629 |  | 8.946768239 |  |
| 36.0738 | 0.69996 | 10.7918813 |  | 2.904062411 |  | 8.586292529 |  |
| 37.1996 | 0.15049 | 50.03226135 |  | 0.622340061 |  | 0.399484317 |  |
| 41.6519 | 0.33159 | 22.3928553 |  | 1.352305032 |  | 1.994256667 |  |
| 44.0059 | 0.44588 | 16.51938475 |  | 1.803815831 |  | 3.664479218 |  |
| 47.5626 | 0.36072 | 20.15344304 |  | 1.440296939 |  | 2.462076231 |  |
| 54.1642 | 0.62538 | 11.31030426 |  | 2.429543085 |  | 7.81720361 |  |
| 56.9277 | 0.07233 | 96.55699468 |  | 0.277448902 |  | 0.107258698 |  |
| 58.4885 | 0.62933 | 11.01449155 |  | 2.395982628 |  | 8.242730336 |  |
| 61.7382 | 0.67139 | 10.15641405 |  | 2.514504837 |  | 9.694361367 |  |
| 64.0155 | 0.35547 | 18.95116234 |  | 1.315237252 |  | 2.784378658 |  |
| 72.7408 | 0.12819 | 49.9004882 |  | 0.450376198 |  | 0.401596955 |  |
| 74.6599 | 0.08728 | 72.37573201 |  | 0.30282016 |  | 0.190903575 |  |

**Table S3:** Size and other lattice parameters of the MZ 3 nanocomposites

| 2θ (degree) | FWHM (degree) | Crystalline size (nm) | Average Crystalline size (nm) | Micro-strain (ɛ × 10^-3^) | Average Micro-strain (ɛ × 10^-3^) | Dislocation Density (δ × 10^-3^) (nm^-2^) | Average Dislocation Density (δ × 10^-3^) (nm^-2^) |
| --- | --- | --- | --- | --- | --- | --- | --- |
| 18.21 | 0.61 | 12.85932948 | 20.98244493 | 2.628090611 | 1.60621766 | 6.0473256 | 3.212189461 |
| 30.13 | 0.20 | 38.35628191 |  | 0.842672525 |  | 0.679715222 |  |
| 35.51 | 0.30 | 25.21965368 |  | 1.246648357 |  | 1.572250578 |  |
| 37.12 | 0.35 | 21.51746666 |  | 1.447736648 |  | 2.159820816 |  |
| 43.15 | 0.61 | 12.1109647 |  | 2.475145588 |  | 6.817772786 |  |
| 53.54 | 0.40 | 17.73208554 |  | 1.558267961 |  | 3.180389892 |  |
| 57.09 | 0.40 | 17.44649445 |  | 1.533170662 |  | 3.285365155 |  |
| 62.68 | 0.30 | 22.61728302 |  | 1.118008957 |  | 1.95487564 |  |

**Table S4:** Size and other lattice parameters of the MZ 4 nanocomposites

| 2θ (degree) | FWHM (degree) | Crystalline size (nm) | Average Crystalline size (nm) | Micro-strain (ɛ × 10^-3^) | Average Micro-strain (ɛ × 10^-3^) | Dislocation Density (δ × 10^-3^) (nm^-2^) | Average Dislocation Density (δ × 10^-3^) (nm^-2^) |
| --- | --- | --- | --- | --- | --- | --- | --- |
| 18.31 | 0.18 | 43.57272741 | 26.11849854 | 0.775393393 | 1.289029286 | 0.526708721 | 2.638479771 |
| 30.15 | 0.23 | 33.35172123 |  | 0.969027864 |  | 0.899007875 |  |
| 35.54 | 0.28 | 27.01879147 |  | 1.163440889 |  | 1.369834692 |  |
| 37.14 | 0.18 | 41.83706602 |  | 0.744506633 |  | 0.571317542 |  |
| 43.2 | 0.41 | 18.01564176 |  | 1.663335251 |  | 3.081062622 |  |
| 53.58 | 0.41 | 17.2965482 |  | 1.596943297 |  | 3.342574665 |  |
| 57.12 | 0.26 | 26.83693733 |  | 0.996418974 |  | 1.388462312 |  |
| 62.72 | 0.41 | 16.54571261 |  | 1.527620687 |  | 3.652826516 |  |
| 71.17 | 0.61 | 10.59134086 |  | 2.164576584 |  | 8.914522995 |  |

**Table S5:** Antibacterial efficacy of various concentrations of MZ 4 against different gram-positive and negative bacteria. Results are presented as mean ± standard deviation

| Tested concentrations of MZ 4 | Zone of Inhibition (in mm) against studied microorganism | | | | | |
| --- | --- | --- | --- | --- | --- | --- |
|  | ***B. subtilis*** | ***B. megaterium*** | | ***S. flexneri*** | ***S. typhimurium*** | ***E.coli*** |
| 300 µg/mL | 18.677±0.577 | | 18±1.00 | 21.667±1.528 | 16.333±1.528 | 28±1.00 |
| 200 µg/mL | 10.667±1.528 | | 7.667±0.577 | 20.333±0.577 | 9±1.00 | 19.667±1.528 |
| 100 µg/mL | 0 | | 0 | 12.667±1.155 | 0 | 14.333±0.577 |

**Table S6:** Antioxidant activity of different grade MZO nanocomposites. Data are presented as mean ± standard deviation (n=3). Different letters (a, b, c, etc.) represented that they are statistically different atp ≤ 0.05 following Tukey’s HSD test.

| Sample Name | Antioxidant activity (IC 50 Value in µg/mL) | | | |
| --- | --- | --- | --- | --- |
|  | **ABTS** | **DPPH** | **SUPEROXIDE** | **NITRIC OXIDE** |
| MZ 1 | 36.845±0.126^a^ | 46.127±1.622^a^ | 62.194±0.180^a^ | 91.487±5.754^a^ |
| MZ 2 | 36.974±0.481^a^ | 47.145±0.681^a^ | 66.456±0.110^b^ | 95.347±6.459^a^ |
| MZ 3 | 57.364±1.647^b^ | 50.781±1.026^b^ | 69.347±1.794^c^ | 114.361±5.349^b^ |
| MZ 4 | 60.784±1.734^c^ | 64.875±1.171^c^ | 75.943±2.456^d^ | 129.473±7.694^c^ |
